# Giant multiferroic effects in topological GeTe-Sb_2_Te_3_ superlattices

**DOI:** 10.1088/1468-6996/16/1/014402

**Published:** 2015-01-13

**Authors:** Junji Tominaga, Alexander V Kolobov, Paul J Fons, Xiaomin Wang, Yuta Saito, Takashi Nakano, Muneaki Hase, Shuichi Murakami, Jens Herfort, Yukihiko Takagaki

**Affiliations:** 1 Nanoelectronics Research Institute, National Institute of Advanced Industrial Science & Technology (AIST), Tsukuba Central 4, 1-1-1 Higashi, Tsukuba 305-8562, Japan; 2 Faculty of Pure and Applied Science, University of Tsukuba, 1-1-1 Tennodai, Tsukuba 305-8573, Japan; 3 Department of Physics & TIES, Tokyo Institute of Technology, 2-12-1 Ookayama, Meguro-ku, Tokyo 152-8551, Japan; 4 Paul-Drude-Institut für Festkörperelektronik, Hausvogteiplatz 5-7, 10117 Berlin, Germany

**Keywords:** multiferroics, magnetoresistance, topological insulator, chalcogenide superlattice, GeTe-Sb_2_Te_3_, phase change memory, spintronics, computer simulation

## Abstract

Multiferroics, materials in which both magnetic and electric fields can induce each other, resulting in a magnetoelectric response, have been attracting increasing attention, although the induced magnetic susceptibility and dielectric constant are usually small and have typically been reported for low temperatures. The magnetoelectric response usually depends on *d*-electrons of transition metals. Here we report that in [(GeTe)_2_(Sb_2_Te_3_)_*l*_]_*m*_ superlattice films (where *l* and *m* are integers) with topological phase transition, strong magnetoelectric response may be induced at temperatures above room temperature when the external fields are applied normal to the film surface. By *ab initio* computer simulations, it is revealed that the multiferroic properties are induced due to the breaking of spatial inversion symmetry when the *p*-electrons of Ge atoms change their bonding geometry from octahedral to tetrahedral. Finally, we demonstrate the existence in such structures of spin memory, which paves the way for a future hybrid device combining nonvolatile phase-change memory and magnetic spin memory.

## 1. Introduction

Although *alternating* electric and magnetic fields are intrinsically coupled, as described by the Maxwell equations, a *static* electric (magnetic) field usually does not induce magnetization (polarization) except in a rather limited—but rapidly growing—class of materials named multiferroics. The idea that for certain classes of magnetocrystalline symmetry there may be ‘a linear coupling between magnetic and electric fields in such media, which would cause, for example, a magnetization proportional to an electric field’ was proposed by Landau and Lifshitz in their classical textbook on theoretical physics over 50 years ago [1]. Since the concept and theory were first proposed, various multiferroic (MF) materials have been discovered and studied, e.g., Cr_2_O_3_ and GaFeO_3_ [2–5]. In multiferroics, the electric dipole, **P**, linearly responds to an external magnetic field **B** as ***Δ*****P** = ***α*****B**, whereas the magnetization, **M**, linearly responds to an external electric field **E**: ***Δ*****M** = ^**t**^***α*****E**, where ***α*** is a 3 × 3 tensor and ^**t**^***α*** is the transposed tensor of ***α***. Multiferroic properties are closely linked to symmetry and can be characterized by their behavior under space and time inversion. Thus space inversion will reverse the direction of polarization **P**, leaving the magnetization **M** invariant, whereas time reversal will change the sign of **M**, preserving the sign of **P**. The important role of symmetry in determining multiferroic properties of crystals makes an important parallel with topological insulators, wherein time reversal symmetry and spatial inversion symmetry also play a crucial role [6]. Although ferroelectric properties are usually determined by the off-center positions of atoms in a structure, ferromagnetic properties typically arise from the presence of transition metal *d*-electrons. Interestingly, the presence of *d*-electrons, which is essential for magnetism, reduces the tendency for off-center ferroelectric distortion [7]. Most MF materials discovered and/or engineered to date are characterized by rather small values of ***α***.

In 2011, a chalcogenide superlattice GeTe-Sb_2_Te_3_ was designed, which led to as much as a 95% reduction in the switching energy of electrical non-volatile phase-change random-access memory (PC-RAM) [8, 9]. Due to the importance of interfaces, the superlattice-based phase-change memory (PCM) was named interfacial phase change memory (iPCM). It was subsequently found that iPCM possesses a giant magnetoresistance (over 2000% at room temperature under a static magnetic field (∼1 kOe)) [10], whereas GeTe-Sb_2_Te_3_ (GST) alloys of the same average composition do not. It should also be noted that previously magnetic effects in PCM were observed only in alloys containing magnetic additives [11–13].

**Figure 1.**
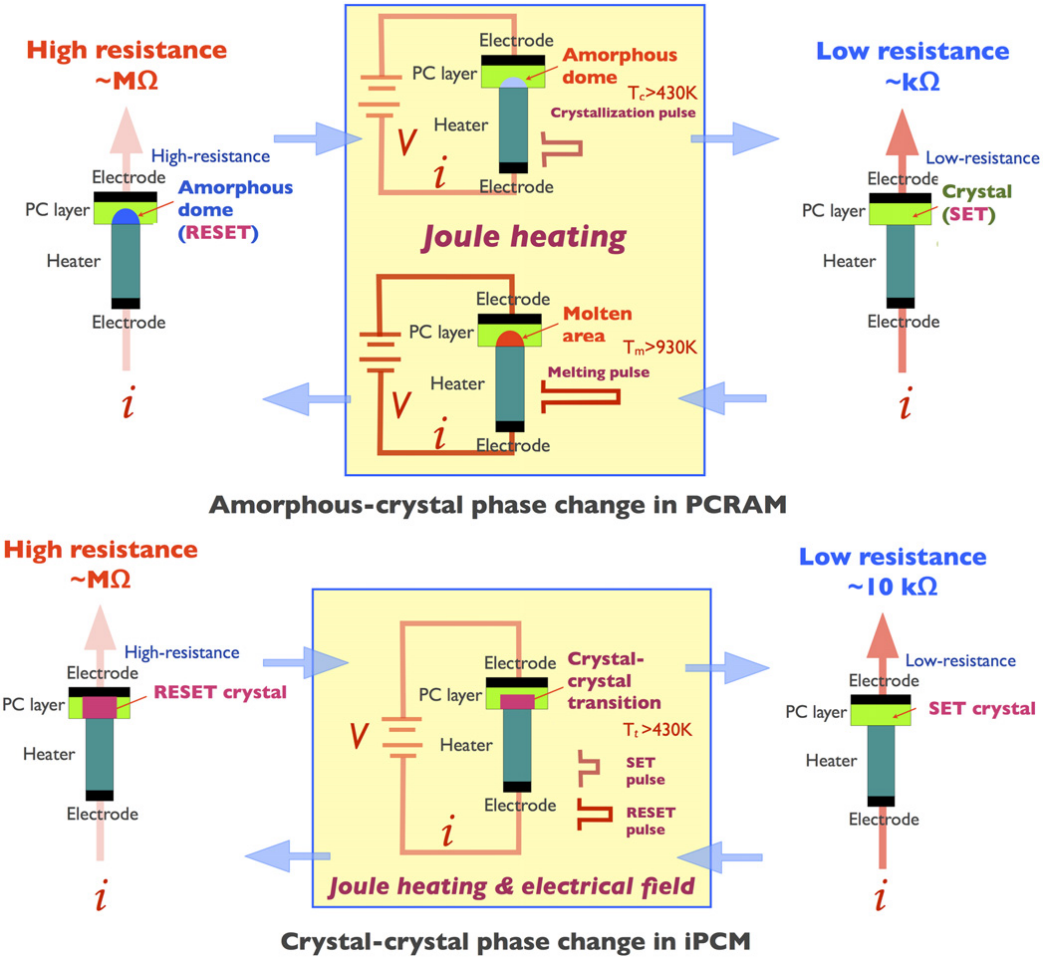
The switching mechanism of PC-RAM and iPCM non-volatile memories. Whereas the former utilizes an amorphous-to-crystalline phase transition, the latter is based on a crystal-to-crystal phase transition. The cell designs can be similar in both cases. The PC and iPCM layer thicknesses are typically about 40 nm, and the bottom electrode (heater) diameter is less than 100 nm. *T*_c_, *T*_m_, and *T*_t_ are the crystallization temperature, melting temperature, and transition temperature, respectively. *V* and *i* are the applied voltage and current. Due to the presence of a melting-free mechanism, the switching energy is reduced by 95% in the iPCM device compared with that of the PC-RAM device using a similarly designed cell platform [9, 10, 16].

PC-RAM, recently commercialized by the world’s largest memory makers, Samsung and Micron, is based on a phase transition between the amorphous and crystalline states in ternary GST alloys, making use of the large property contrast between the two phases. The contrast is associated with the contrasting bonding nature between the constituent atoms in the structures. When a voltage exceeding a certain value is applied to a device in the high-resistivity amorphous (RESET) state, it switches to the low-resistivity crystalline (SET) state. During the SET pulse, the GST is heated above the crystallization temperature, whereas the RESET pulse melts the material, reverting it to the amorphous phase with large entropic losses [14–16]. Pulses with typical durations of ∼100 ns and ∼500 ns are used for the RESET and SET processes, respectively. In contrast, in iPCM, which has the structure of a short-period [(GeTe)_*l*_(Sb_2_Te_3_)_*m*_]_*n*_ *superlattice* (where *l, m*, and *n* are integers), where GeTe and Sb_2_Te_3_ share a common growth axis, the [111] direction of the rhombohedral GeTe layer and the [111] axis of the rhombohedral (A7) Sb_2_Te_3_ layer, being parallel to each other and normal to the substrate surface, is crystalline in both the SET and RESET states [9]. Importantly, even sputtered iPCM devices have a strong preferred [111] growth direction and exhibit high-quality interfaces [17]. The switching mechanisms of PC-RAM and iPCM are summarized in figure 1.

In a previous work [16, 18], we examined, using **ab initio** molecular dynamics, possible structures of iPCM in the SET and RESET states. The results of **ab initio** molecular dynamics using constant temperature and constant pressure ensembles (NPT ensembles) for various studied structures are shown in figure S1. It was found that the structures with the lowest energies at the growth temperature (∼520 K) consist of two buckled GeTe layers sandwiched by quintuple layers (QLs) of Sb_2_Te_3_. The stacking sequences within the GeTe block are (-*v*-Te-Ge-Te-Ge-) in the SET and (-*v-*Te-Ge Ge-Te-*v*-) in the RESET state, where *v* indicates interatomic distances exceeding 3.1 Å, often referred to as vacancy layers or van der Waals gaps.

**Figure 2.**
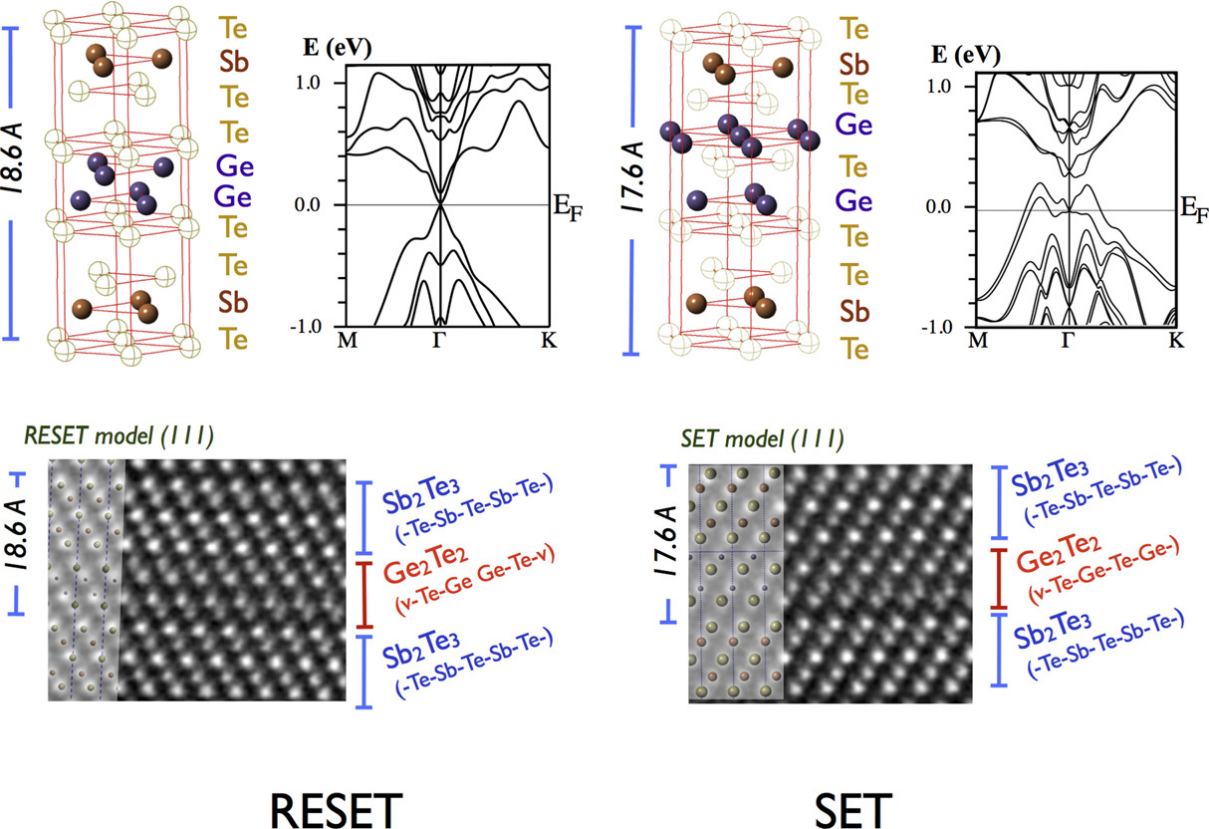
The upper panel shows the RESET and SET models and the corresponding bulk band structures, and HAADF TEM images of the [(GeTe)_2_/(Sb_2_Te_3_)_1_]_20_ iPCM (∼40 nm thick) grown on Si(111).

The structures of the SET and RESET states are shown in the upper panel of figure 2 together with the corresponding band structures. One can see that the RESET phase has a Dirac-like density of states. This fact, combined with the calculated topological invariant *Z*_2_ = 1, suggests that the RESET state is a strong topological insulator protected by spatial inversion and time reversal symmetries [19]. The SET phase, on the other hand, has a gap due to the breaking of spatial inversion symmetry, resulting in spin-split band structures. The differences between the two phases were clearly observed using high-angle annular dark field transmission electron microscopy (HAADF-TEM), as shown in figure 2. (See the supplementary material for details.) It is noted that the obtained HAADF-TEM images of real iPCM films are in good agreement with the results of earlier simulated models for the SET and RESET states [18, 19]. When one Ge layer is swapped with the adjacent Te layer by an external stimulus such as temperature or electric field, the RESET state transforms into the SET state and vice versa [18]. The change of the electric dipole moment (dielectric constant) induced by the phase transition generates large optical and electrical contrast [9].

iPCM devices have another important attribute. A number of groups have recently argued that, depending on the thicknesses of individual blocks, iPCM can be either a strong topological insulator (TI) with a single Dirac cone at the *Γ* point [19] or a Dirac semimetal composed of alternating layers of a narrow gap normal insulator (NI), GeTe, and a three-dimensional topological insulator (3D-TI), Sb_2_Te_3_ [18–21]. In addition, recent **ab initio** simulations of bulk GeTe have predicted a giant electric field-induced Rashba effect, which also originates from the *p*-electrons of Ge atoms [22]. These properties of iPCM offer previously unexplored possibilities for multiferroics and spintronics.

In this work, breaking either spatial inversion or time reversal symmetries or both in the topological insulating phase of the superlattices, we describe the magnetoelectric effects when external electric and magnetic fields are applied to iPCM. Subsequently we discuss the origin of magnetism in the absence of magnetic *d*-electrons and, finally, demonstrate the applicability of iPCM for spin-storage devices.

## 2. Experimental procedures

### 2.1. Fabrication of iPCM devices

iPCM films were fabricated on Si wafers using a helicon-wave sputtering system that features a large (200 mm) target–substrate separation. The individual layers composing the (GeTe)_*x*_/(Sb_2_Te_3_)_*y*_ structure were fabricated using GeTe and Sb_2_Te_3_ composite targets (2-inch diameter) using an automated shutter control system with a substrate temperature of about 520 K and pressures less than 0.5 Pa. The thicknesses of the GeTe and Sb_2_Te_3_ blocks were 0.4 nm and 1.0 nm, respectively. To ensure strong crystalline orientation, a 5 nm-thick Sb_2_Te_3_ layer was deposited prior to the fabrication of iPCM films. As a consequence, all the sub-layers were crystalline in the as-deposited state and exhibited a strong preferred crystallographic orientation toward the <111> direction (see [8, 9] for more details). For electrical and magnetic measurements on real devices, the iPCM film was deposited on a base device with a TiN rod electrode (70 nm diameter) embedded in a silicon nitride layer. A 40 nm-thick TiN layer was deposited on the iPCM film to serve as an electrode.

The structure of the deposited iPCM was studied using high-angle annular dark field (HAADF) transmission electron microscopy (JEOL, JEM-ARM200F). The samples were prepared using ion milling. The images were observed using an electron beam with a diameter of 0.2 nm and an accelerating voltage of 200 kV in combination with fast Fourier transform (FFT) analysis.

In addition, we developed an alternative device design that is more suitable for hybrid memory. In this design, the superlattice film was deposited onto a flat and cleaned (dry-etched) Si wafer, and successively a sequence of ZnS-SiO_2_, ferromagnetic TbFeCo, and ZnS-SiO_2_ layers, 10 nm thick each, were deposited. By exposure to a focused laser pulse (*λ* = 405 nm), the ZnS-SiO_2_/TbFeCo/ZnS-SiO_2_ layers inter-diffused, resulting in the formation of an electrical contact pillar [23] with a diameter of about 400 nm directly connected to both the ferroelectric layer and the phase-change film. Finally, a TiN electrode was deposited on top. When a voltage is applied to such a device, electrons from the ferroelectric TbFeCo layer participate in the current flow. When an external magnetic field is applied to the device, spin-polarized electrons can be injected from the TbFeCo into the phase-change layer, which allows one to obtain different states when the device is SET with and without a magnetic field.

### 2.2. Device measurement

The Hall resistance and coefficient of the iPCM were measured maintaining a constant current of 10 *μ*A by a ResiTest 8400 unit (Toyo Co.) using a van der Pauw configuration. The current flowed in plane between two selected contacts among four probes, whereas the magnetic field was applied normal to the plane. Each measurement was repeated ten times with good reproducibility. As a substrate, an undoped high-resistance Si (100) wafer was used. Before the iPCM film measurement, we confirmed that the background signal from the substrate was negligible.

Resistance–voltage (R–V) curves were taken using a programmable pulse generator with a 300 ns pulse for which the voltage was increased in 50 steps. An iPCM device and a control PC-RAM device with identical architectures were compared. In the iPCM device, the threshold voltages were 0.85 V for SET and 1.5 V for RESET, whereas those in the control PC-RAM device were 1.0 V for SET and 3.5 V for RESET [10]. The iPCM switched from the SET to the RESET state at 0.20 mA, whereas the control device did not switch until 1.1 mA. The magnetic field in the device measurements was applied using three different (0.8 kOe, 1.0 kOe, and 1.2 kOe) 1 mm-thick flat, square magnets with areal dimensions of 10 mm × 10 mm.

### 2.3. Simulation of band structures

The electronic band structures of iPCM films shown in figure 2 were simulated in two steps using two *ab initio* simulation codes: CASTEP and WIEN2K. iPCM models were first built and relaxed at 0 K using the plane wave code CASTEP with a GGA exchange correlation term using the Perdew–Burke–Ernzerhof 1996 (PBE) function. A 4 × 4 × 1 Monkhorst–Pack grid was used for integration, and ultrasoft pseudopotentials were used with a cutoff energy of 230 eV [24, 25]. After 0 K Broyden geometrical optimization, the CASTEP-relaxed models were transferred to WIEN2K [26], which allows one to include spin–orbit coupling (SOC) effects. Wien2K is an all-electron code that uses a linearized augmented plane wave + local orbital (LAPW + lo) basis within density-functional theory using the PBE exchange correlation. The same Monkhorst–Pack grid of 4 × 4 × 1 was used for integrations in the Brillouin zone, and an R_*x*_K_max_ = 7.0 value was used for the plane wave component of the plane-wave basis used between augmentation spheres.

In **ab initio** molecular dynamics, on the other hand, constant temperature and constant pressure ensembles (NPT ensemble) were used at 0 GPa, using a cutoff energy of 170 eV. The smaller cutoff energy was used to increase the simulation speed and did not have a strong effect on the result. The free energy changes were simulated at a number of different temperatures to identify the phase transition temperature between the SET and RESET states.

## 3. Results and discussion

### 3.1. Magnetoelectric response of an iPCM device

**Figure 3.**
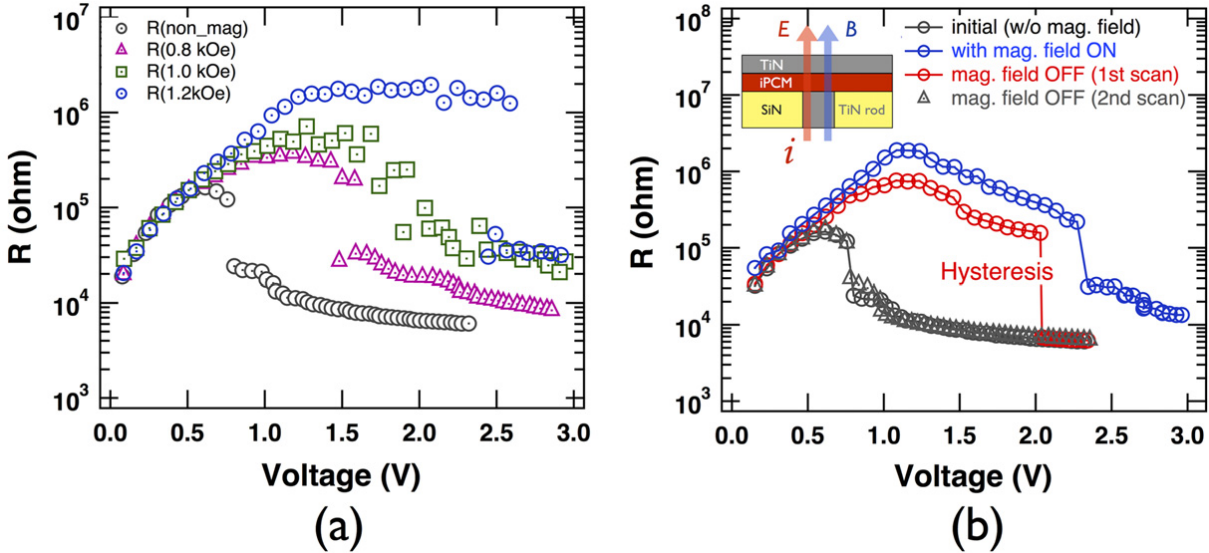
(a) Magnetoresistance under different magnetic field strengths 0.8, 1.0, and 1.2 kOe and (b) the associated resistance hysteresis in a typical iPCM [(GeTe)_2_/(Sb_2_Te_3_)_4_]_8_ device (inset) at room temperature. Magnetoresistance hysteresis occurs after the electric field is turned off with a magnetic field still present. The black circles indicate the initial resistance without an applied magnetic field, the blue circles indicate resistance values with a magnetic field, the red circles indicate the first R–V curve after removal of the magnetic field, and finally the black triangles correspond to the second R–V cycle after the magnetic field was turned off. In each process, the initial and terminated states were both RESET.

Figure 3(a) shows R–V curves of an iPCM [(GeTe)_2_/(Sb_2_Te_3_)_4_]_8_ device when external magnetic fields of different intensities are applied normal to the interfaces. Without an external magnetic field, the transition to the SET phase occurs at the same threshold voltage as in the alloy of the same average composition (cf figure 6 of [18]) with an important difference: the resistance of iPCM increases by an order of magnitude for voltages between 0 V and 0.8 V (the threshold voltage), whereas the resistance of the composite amorphous phase in PC-RAM remains constant (or gradually decreases, which has been explained by the Frenkel–Poole mechanism [15]). Upon increasing the magnetic field to 1.2 kOe, the resistance in the RESET phase increases by two orders of magnitude between 0 V and 2.5 V and then suddenly drops to the low-resistance SET phase. The minimum magnetic field required for the magnetoelectric effect was ∼0.8 kOe, and lower fields had a negligible effect (not shown). It should also be noted that the resistance curves in the range between 0 and 0.5 V almost completely overlap regardless of the magnitude of the magnetic field. Therefore, it can be surmised that the unusual resistance increase in this voltage range is due to an intrinsic magnetic field induced by the applied electric field, which is equivalent to an external magnetic field of ∼0.8 kOe, after which the external magnetic field starts to have an effect on the threshold voltage. This implies that the ***α*** of ***Δ*****M** = ^**t**^***α*****E** in the RESET phase has diagonal elements since both the induced magnetization and the applied electric field have the same direction normal to the surface.

Of special interest is the device behavior in subsequent cycles (high resistance state -> low resistance state -> high resistance state). If the device is in the SET state (the electric field is turned off) in the presence of a magnetic field followed by removal of the magnetic field, in the subsequent (i.e., second) cycle the R–V characteristics remain essentially unchanged (figure 3(b), red curve), namely, the threshold voltage for the second cycle is similar to the R–V curve for the first cycle in the presence of the magnetic field; i.e., the device displays a memory of the magnetic field applied during the previous cycle. In the third cycle (black curve), however, the memory of the magnetic field is erased: the R–V curve reverts to its original behavior before the application of the magnetic field. It should be noted that the difference between the R–V curves at 1.2 kOe shown in figures 3(a) and (b) is due to the fact that the magnetic field was increased in steps (0.8 to 1.0 to 1.2 kOe) in figure 3(a) and was set to 1.2 kOe from the beginning in figure 3(b). Reproducibility of this cycle was confirmed for several identical devices. It should be noted that no magnetic dopants or other impurities were included in the iPCM.

**Figure 4.**
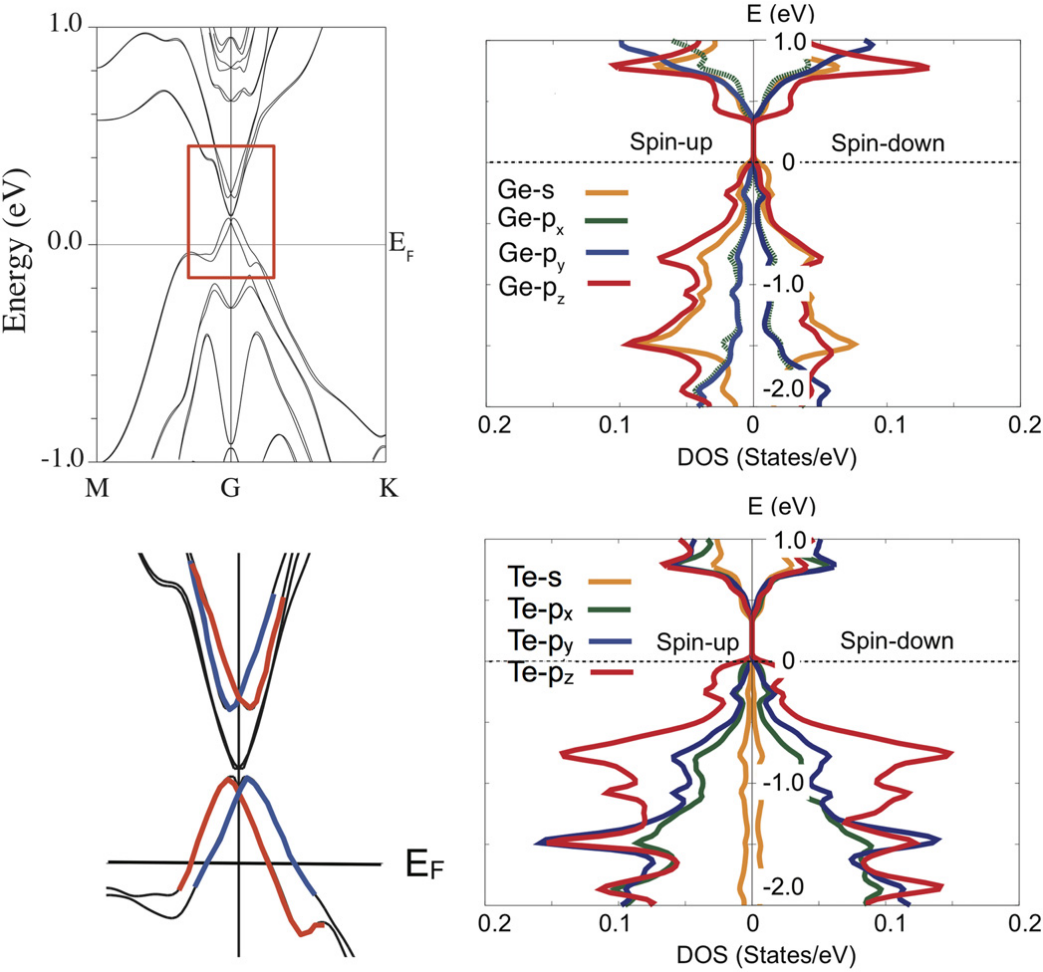
The band structure for the iPCM-RESET phase under an external electric field (0.51 V nm^−1^) at 0 K (upper left), the band structure details around the *Γ* point (lower left), and the corresponding spin-polarized density of states for Ge (upper right) and Te (lower right) atoms.

When an external electric field of 0.001 electrostatic units (a.u.) (corresponding to 0.51 V nm^−1^) is applied normal to the iPCM film in a simulation including SOC, the Dirac cone is broken and a gap opens as shown in figure 4 (left). Because the conductivity of Sb_2_Te_3_ is higher than that of GeTe, the voltage drop is mainly across the GeTe block (∼0.9 nm). The total voltage applied to the structure with eight repeat units (in the experiment we used eight repeats; hence we also consider eight repeats in the simulation) becomes 3.7 V. This value is four times larger than that obtained experimentally (0.85 eV), but it should be kept in mind that even though the simulations were performed at 0 K, in the experiment the device contained a TiN heater rod, and an increase in temperature in the presence of a current is likely to facilitate the breaking of spatial inversion symmetry. In the foregoing simulation, the maximum energy difference, *Δ*E_tvb_, between the spin-up and spin-down bands of 0.07 eV was obtained at *k* ∼ 0.06 Å^−1^ (the M-point corresponds to *k* = 0.76 Å^−1^). In a different simulation (not shown) we found that a displacement of two Ge atoms from the stable position by 0.1 Å and 0.2 Å along the *c*-axis caused a splitting *Δ*E_tvb_ between the spin-up and spin-down bands of 0.09 eV and 0.24 eV, respectively, whereas the band gap, *Δ*E_gap_, simultaneously expanded to 0.15 eV and 0.35 eV. These results support the assumption that the superlattice becomes more insulating through the breaking of spatial inversion. In the presence of the Rashba effect, the spin density of states (SDOS) for two opposite spin orientations is no longer degenerate and originates from Ge *p*-electrons (figure 4 (right)).

The Rashba parameter can be roughly estimated from the simulation of electric field effects, shown in figure 4 (left panel) to be about 1.2 eVÅ. This value is comparable to the values found for other materials with giant Rashba splitting such as two-dimensional Bi-Ag alloy thin layers on Ag(100) or (111) surfaces (3.6 eVÅ and 3.1 eVÅ, respectively) [27, 28] and also to that of Pb/Ge(111) (1.5 eVÅ) [29]. Therefore, the value of 1.2 eVÅ obtained from the simulation results is a reasonable value. From the NTP ensemble simulations (supplemental figure S1, available at stacks.iop.org/STAM/16/014402/mmedia), it was found that in the iPCM, the Rashba parameter depends not only on the strength of the external electric field normal to the film but also on the temperature. Since the phase transition temperature between the RESET and SET states is around 420 K, the value of the Rashba parameter is likely to be larger.

### 3.2. Two-dimensional transport properties of iPCM films

It is speculated that a pair of spin states exists where the spins are oriented in plane and antiparallel to each other. This is strongly supported by the band structure of the RESET phase in figure 2, which has a single Dirac cone at the *Γ* point. It should be noted that the unit cell of the iPCM RESET phase satisfies both spatial inversion symmetry and time reversal symmetry, although the cone is spin-degenerate. Two spin states exist in plane to maintain time reversal symmetry, i.e., *E*(*k*, →) = *E*(−*k*, ←), where *k* and the arrows are the electron momentum and spin directions.

**Figure 5.**
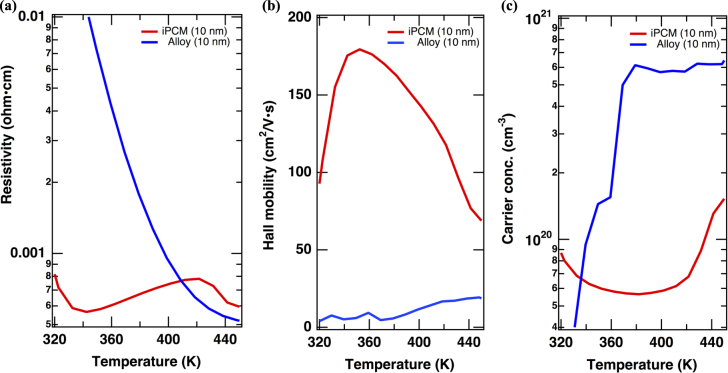
Temperature dependence of (a) the resistivity, (b) Hall mobility, and (c) carrier concentration of the [(GeTe)_2_(Sb_2_Te_3_)_1_]_2_ structure (red) and the control alloy with the same composition ratio (blue). The Hall properties of (a)–(c) were measured under a 5 kOe magnetic field.

We further studied transport properties using Hall effect measurements on iPCM films. In this case the electric field is applied in plane, whereas the magnetic field (5 kOe) is applied normal to the plane. If the iPCM RESET phase does have a Dirac cone, two-dimensional metallic transport should be observed. Figure 5 shows the results obtained for an iPCM [(GeTe)_2_(Sb_2_Te_3_)_1_]_2_ film fabricated at 520 K with a control film of GST alloy fabricated with the same film thickness and at the same temperature. After the initial drop, the resistivity (ρ_*xx*_) of the RESET phase increased to 7.8 × 10^−4^ *Ω* cm at ∼420 K, at which point the SET phase of the iPCM film was established. Therefore, when the electric field is applied in plane, the RESET phase of the iPCM film behaves like a metal at temperatures between 350 K and 420 K. It should be noted that in the control GST alloy film with the same average composition, the resistivity monotonically decreased as expected for a semiconductor. At the same time, the Hall mobility steeply increased, reaching a value of ∼180 cm^2^ V-s^−1^ at ∼350 K, and then monotonically decreased, whereas the carrier concentration of 5.7 × 10^19^ cm^−3^ was essentially unchanged until the phase transition (apart from the initial drop). The results from the iPCM film are crucially different from the behavior of the alloy of the same average composition of our control sample and other results reported in the literature [15]. We have also checked the magnetization of the films using SQUID at 2 K and 300 K (see supplemental figure S2). As expected, the magnetization of the RESET phase was negligible (within the experimental errors) because of the spin-band degeneracy. These results support the existence of a metallic or gapless state with high mobility in the RESET phase. The change in the Hall mobility and carrier concentration with temperature is interpreted as arising from internal stresses which generate optimal conditions for the formation of a proper TI or Dirac semimetal phase, reflecting the two-dimensional nature [19, 20].

### 3.3. Role of *p*-electrons in the iPCM films

An external electric field induces, via the Rashba effect, a band opening similar to the result [22] reported for bulk GeTe, and we speculate that the magnetic properties appearing in the Hall measurement are due to the presence of thin GeTe layers. As shown in figure 4, under an applied electric field the spin-polarized electron densities of states in Ge and Te are different for spin-up and spin-down states. For this reason, when an electric field is applied and current flows, the iPCM RESET structure can have a magnetic moment. The magnetic moment disappears when the electric field is turned off and the structure reverts to the original symmetrical structure.

Ge atoms in GeTe, such as those present in iPCM, were shown to possess lone-pair electrons [30], a situation that is not uncommon for elements containing paired *s*-electrons that formally do not participate in nominal *σ*-electron (bonding) formed using *p*-electron orbitals [31] but become stereochemically active when the bonding angles deviate from 90°. Such lone-pair electrons have been considered as the origin of ferroelectricity in GeTe and related materials [32, 33]. Real GeTe crystals are characterized by a high concentration of Ge vacancies [34] (which have a very low formation energy [35]), generating a change in the density of states. In particular, Ge atoms nearest the vacancy may have lower electron density on the lone-pair orbital, in the limit changing it to an unpaired *p*-electron (supplemental figure S3). In the RESET phase, where the two GeTe buckled layers have an antiferroelectric arrangement of dipole moments with the adjacent Ge planes, interaction of the unpaired electrons from the neighboring layers results in a net zero spin moment.

**Figure 6.**
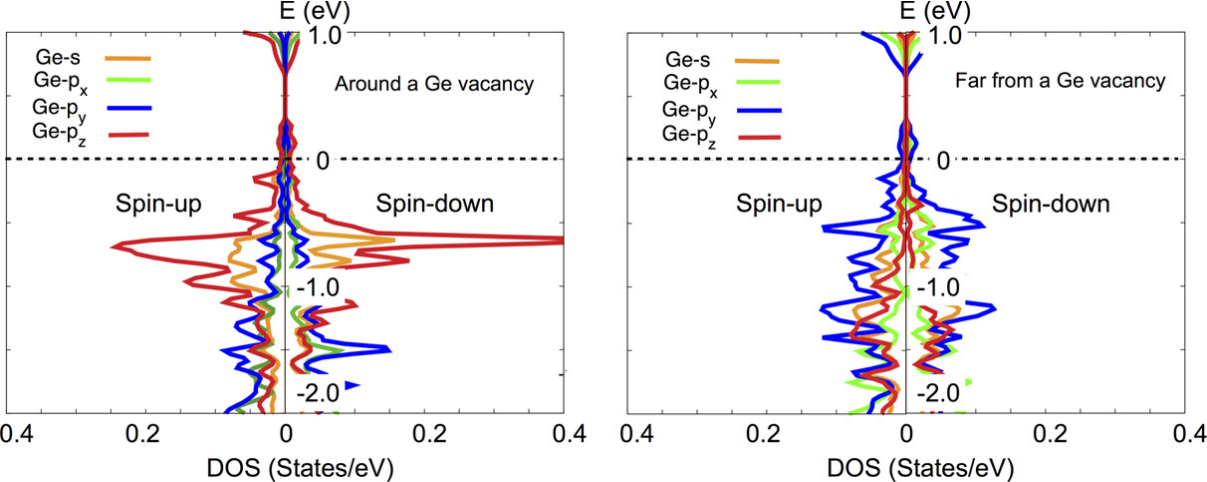
Spin-polarized density of states for a Ge atom near a Ge-site vacancy (left) compared with a Ge atom located away from the vacancy (right) in the iPCM-SET phase.

However, the situation with the iPCM SET phase is different. Since the GeTe block loses spatial inversion symmetry, spins located at Ge atoms in different layers do not cancel each other. Indeed, as shown in figure 6 (left), Ge *p*-electrons around the vacancy exhibit a large asymmetry between the spin-up and spin-down configurations, the situation being significantly different from Ge atoms located away from vacancies (figure 6 right) and similar to the case of a graphene ribbon where zigzag edges becomes ferromagnetic due to unpaired electrons localized at the edge C atoms [36, 37]. The magnetization of the SET phase when the spatial inversion was broken was recently confirmed experimentally using magneto-optical Kerr rotation measurement [38]. It was reported that the Kerr rotation was observed only in the high-temperature SET phase (>420 K) with unusual mirror-symmetric curves with a relatively large angle (∼0.3°) [38].

It should also be noted that the magnetoelectric response of iPCM is unusually strong. We believe this is caused by the rather special origin of magnetism in these structures. Although typically ferromagnetic properties are due to the presence of transition metal *d*-electrons that tend to reduce the off-centering of atoms like Bi or Pb that are responsible for ferroelectricity, in iPCM, the ferromagnetic properties arise from unpaired electrons located on Ge atoms along with the ferroelectric properties caused by off-centering of the same Ge atoms; i.e., *both the ferroelectricity and ferromagnetism in iPCM are due to the same atomic species and p-electrons*.

### 3.4. Proposal of topological switching random access memory (TRAM or TSRAM)

To implement the observed spin storage practically, we developed a special device design, in which there is an additional ferromagnetic TbFeCo layer that can act as a spin-injection source. When voltage is applied to this structure and current flows under an applied magnetic field, spins in the TbFeCo layer, aligned by the magnetic field, are injected into the superlattice through the conducting pillar.

**Figure 7.**
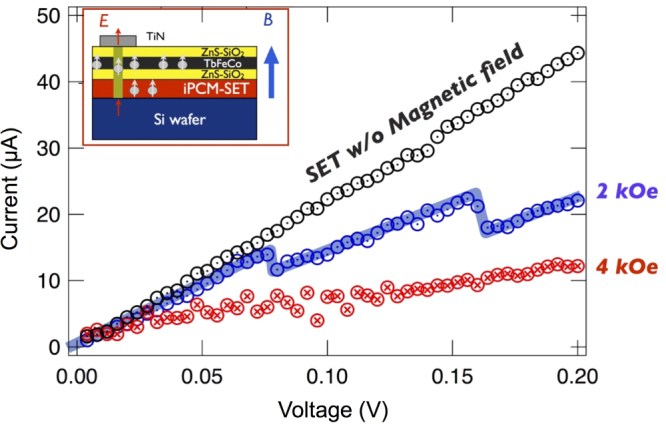
I–V curves from the iPCM-SET phase with and without a magnetic field (2 kOe and 4 kOe) for the spin-storage device (inset) at room temperature, using iPCM [(GeTe)_2_(Sb_2_Te_3_)_4_]_8_.

Figure 7 shows I–V curves for this structure (shown in the inset) SET either with or without a magnetic field. The curves shown clearly demonstrate a magnetoresistance response depending on the strength of the magnetic field. In particular, at intermediate field strength (2 kOe), an I–V curve with several steps was observed, which supports practical applicability for multilevel magnetoresistance memory devices.

## 4. Conclusions

In this work, we described the important role of the topological phase transition in [(GeTe)_2_(Sb_2_Te_3_)_*l*_]_*m*_ superlattices due to the breaking of the spatial inversion and time reversal symmetries when external electric and magnetic fields are applied. Through the combined use of experiments and **ab initio** simulations, we demonstrated that superlattices with appropriately chosen thicknesses of individual layers are high-temperature multiferroic materials. We argue that the giant magnetoelectric response of iPCM is due to the fact that, different from most other cases, both ferroelectric and ferromagnetic properties are associated with the same atomic species, namely Ge atoms. The demonstrated spin storage, TRAM, in iPCM-based devices opens up new avenues for use by future nonvolatile storage memory in the form of new hybrid devices combining the attributes of PCM, MRAM, and topological insulators.
